# Supplementary material for: A highly parallel strategy for storage of digital information in living cells
Source: BMC Biotechnol. 2018 Oct 17;18:64. doi: 10.1186/s12896-018-0476-4 (PMC6191901; doi:10.1186/s12896-018-0476-4)
Supplement: Supplementary file 1 — Supplementary information. (DOCX 34 kb) [file 12896_2018_476_MOESM1_ESM.docx]

# Additional file

## Supplementary tables and figures

## Supplementary text

### S1. General estimates of error tolerance

### S2. Estimated information content in other publications

## References

## Supplementary text

### S1. General estimates of error tolerance

In any round trip write-store-read experiment, we can group errors into three classes:

- Errors arising during synthesis, with per nucleotide rate $s'=1-s$
- Errors arising during storage, with per nucleotide rate $r'=1-r$
- Errors arising during sequencing, with per nucleotide rate $q'=1-q$

These grouping are advantageous in that the parameters of one can often be manipulated independently from the other two. For instance:

- Synthesis errors can be improved by using more sophisticated synthesis technology or producing shorter pieces of DNA
- Storage errors are influenced strongly by duration of storage, storage medium and temperature
- Sequencing errors depend on choice of sequencing technology and prep

For any given position in the encoded data, the fraction of DNA molecules in the synthesized pool that carry the correct nucleotide at that position will be $s$. After storage, this proportion will fall to $s\cdot r$. The proportion of sequencing reads covering this position that also correctly report the unaltered bases will be $p=s\cdot r\cdot q$.

#### Correcting errors by majority consensus

After processing sequencing data and obtaining $c$ reads covering each position, the probability of a majority of reads reporting the correct base is given by the binomial distribution $P=B_{CDF}(c,k,p')$ representing probability of no more than $k$ successes from $n$ trials with probability of success $p'=1-p$, where $k$ is $(c+1)/2$ rounded down.

Picking a modest estimate of $c=5$, we get $k=3$. In our simulations we have determined that redundancy-based error correction can recover from an error rate of up to $P=0.01$ in the final assembled consensus sequence. This implies a maximum threshold for the round trip error rate $p'\approx0.10$. If coverage is increased slightly to $c=10$, then $k=4$ and $p'\approx0.15$. For much higher but still reasonable $c=50$; $k=24$ and $p'\approx0.33$. Note, however, that this does not include the effect of errors on assembly.

#### Real world estimates of error rates

Synthesis errors tend to be on the order of 1% to 0.01% [1], although they are often strongly influenced by certain parameters such as desired fragment length (shorter pieces have less error) and yield (there is a trade-off between fidelity and total yield). We can take $s'=0.01$ as an estimate easily achieved by even older technology. However it is clear that much higher synthesis fidelities can be achieved - for example, in our case encoded information is composed solely of a limited repertoire of n-mers, so synthesizing DNA from pre-manufactured n-mers instead of monomers would not be technically challenging and could improve the segment length several fold, albeit with the potential to introduce segmental substitution error.

The experiment of Grass et al. [2] can be interpreted as a measurement of $r'$. They report per nucleotide error rates on the order of 1% after simulated storage for 2000 years. Thus we can take $r'=0.01$

Sequencing error rates can vary substantially depending on the particular equipment and protocol used. Notably, statistical properties of the sequence (such as repetitiveness) also have a strong effect, and our encoding method minimizes problematic sequence features. Nevertheless, commonly reported rates are on the order of 0.1-1% [2] and from this we can take $q'=0.01$.

Based on the above, we can estimate $p=s.r.q=0.99\cdot0.99\cdot0.99\approx0.97$ and correspondingly $p'=0.03$ for a reasonable expectation of the error rate in practice, after storage for many centuries. This is well below the maximum even for 5x coverage, which is $p'=0.10$. Thus, high-fidelity storage appears to be very feasible using the method we propose, even on extremely long timescales and modest technical resources.

Note that for the above calculations provide an estimate of per-nucleotide errors, but it is difficult to precisely calculate true error rates in actual usage due to the many, sometimes unpredictable factors involved. For the above estimates, it is assumed that:

1. Assembly is successful. The likelihood of this is explored in the sections of our main manuscript titled “Library construction and long-term packet-wise retention” and “Simulated recovery of information with packet loss”.
2. There is no block-wise synthesis error (as would be the case if packets were synthesized from n-mers rather than individual monomers, for instance).
3. There are no systematic errors or jackpot effects. Our main manuscript, in the section “Encoding of digital data into DNA”, discusses the features of our codec which enable mitigation of systematic errors.

Therefore, real world performance will likely be lower than these estimates.

### S2. Details of data in Table 6

#### Bancroft 2001

The phrase encoded was “IT WAS THE BEST OF TIMES IT WAS THE WORST OF TIMES IT WAS THE AGE OF FOOLISHNESS IT WAS THE EPOCH OF BELIEF” [4], which is 106 characters long. The authors specify that they mapped values to uppercase letters and a space, a total of 27 possible characters. In this case, Shannon information would be $107.\log_{2} 27=509$ bits.

#### Church 2012

The publication reported encoding of 5.27 million bits in total. This was stored on 54898 sequences, each 159 bp long, for a total of 8728782 bp. [5]

#### Goldman 2013

The numbers for total bits of information and total bases used were taken as reported in the publication. [6]

#### Grass 2015

It was reported that 83 kbytes of information was encoded ($83000.8=664000$ bits) and 4991 segments of DNA, each 158 long, were produced ($4991.158=788578$ bp of DNA). [2]

#### Yazdi 2015

While the total raw input information was given as 17 kbytes, this study encoded information using a dictionary of words rather than traditional byte-characters. We have therefore taken their own figure of 23196 bits of information for the word-based encoding (Table S1). [7]

It was reported that the total of bases produce was 32 kbp.

#### This publication

The Hamming.jpeg image was 10478 bytes, or $8.10478=83824$ bits. The encoded DNA produced from this was 111192 bp long.

## References

| [1] | S. Kosuri and G. M. Church, "Large-scale de novo DNA synthesis: technologies and applications.," *Nature methods,* vol. 11, no. 5, pp. 499-507, 5 2014. |
| --- | --- |
| [2] | R. N. Grass, R. Heckel, M. Puddu, D. Paunescu and W. J. Stark, "Robust chemical preservation of digital information on DNA in silica with error-correcting codes.," *Angewandte Chemie (International ed. in English),* vol. 54, no. 8, pp. 2552-2555, 2 2015. |
| [3] | T. C. Glenn, "Field guide to next-generation DNA sequencers.," *Molecular ecology resources,* vol. 11, no. 5, pp. 759-769, 9 2011. |
| [4] | C. Bancroft, T. Bowler, B. Bloom and C. T. Clelland, "Long-term storage of information in DNA," *Science,* vol. 293, pp. 1763-1765, 2001. |
| [5] | G. M. Church, Y. Gao and S. Kosuri, "Next-generation digital information storage in DNA," *Science,* vol. 337, pp. 1628-1628, 2012. |
| [6] | N. Goldman, P. Bertone, S. Chen, C. Dessimoz, E. M. LeProust, B. Sipos and E. Birney, "Towards practical, high-capacity, low-maintenance information storage in synthesized DNA," *Nature,* vol. 494, pp. 77-80, 2013. |
| [7] | S. M. H. T. Yazdi, Y. Yuan, J. Ma, H. Zhao and O. Milenkovic, "A rewritable, random-access DNA-based storage system," *Scientific reports,* vol. 5, p. 14138, 2015. |
